# The floral transition is not the developmental switch that confers competence for the *Arabidopsis* age-related resistance response to *Pseudomonas syringae* pv. *tomato*

**DOI:** 10.1007/s11103-013-0083-7

**Published:** 2013-05-31

**Authors:** Daniel C. Wilson, Philip Carella, Marisa Isaacs, Robin K. Cameron

**Affiliations:** Department of Biology, McMaster University, 1280 Main St West, Hamilton, ON L8S 4L8 Canada

**Keywords:** *Arabidopsis*, *Pseudomonas syringae*, Age-related resistance, Developmental resistance, Flowering, Photoperiod

## Abstract

Age-related resistance (ARR) is a plant defense response characterized by enhanced resistance to certain pathogens in mature plants relative to young plants. In *Arabidopsis thaliana* the transition to flowering is associated with ARR competence, suggesting that this developmental event is the switch that initiates ARR competence in mature plants (Rusterucci et al. in Physiol Mol Plant Pathol 66:222–231, 2005). The association of ARR and the floral transition was examined using flowering-time mutants and photoperiod-induced flowering to separate flowering from other developmental events that occur as plants age. Under short-day conditions, late-flowering plant lines *ld*-*1* (*luminidependens*-*1*), *soc1*-*2* (*suppressor of overexpression of co 1*-*2*), and *FRI* ^+^ (*FRIGIDA*) displayed ARR before the transition to flowering occurred. Early-flowering *svp*-*31*, *svp*-*32* (*short vegetative phase*), and Ws-2 were ARR-defective, whereas early-flowering *tfl1*-*14* (*terminal flower 1*-*14*) displayed ARR at the same time as Col-0. While *svp*-*31*, *svp*-*32* and Ws-2 produced few rosette leaves, *tfl1*-*14* produced a rosette leaf number similar to Col-0, suggesting that the development of a minimum number of rosette leaves is necessary to initiate ARR competence under short-day conditions. Photoperiod-induced transient expression of *FT* (*FLOWERING LOCUS T*) caused precocious flowering in short-day-grown Col-0 but this was not associated with ARR competence. Under long-day conditions *co*-*9* (*constans*-*9*) mutants did not flower but displayed an ARR response at the same time as Col-0. This study suggests that SVP is required for the ARR response and that the floral transition is not the developmental event that regulates ARR competence.

**Electronic supplementary material:**

The online version of this article (doi:10.1007/s11103-013-0083-7) contains supplementary material, which is available to authorized users.

## Introduction

The outcome of a plant-pathogen interaction often depends on the developmental stage of the plant (Agrios 2005). Under short-day conditions (9 h light), young (3- to 4-week-old) *Arabidopsis* are susceptible to the bacterial pathogen *Pseudomonas syringae* pv. *tomato* (*Pst*), as indicated by the presence of disease symptoms and high *in planta* bacterial growth. In contrast, mature plants (>5 weeks old) are typically asymptomatic and show a 10- to 100-fold reduction in bacterial growth (Kus et al. 2002). *Arabidopsis* age-related resistance (ARR) also confers protection against the oomycete *Hyaloperonospora arabidopsidis* (Rusterucci et al. 2005). ARR has been observed in many plant species and the mechanisms involved appear to differ widely (Reviewed in Develey-Rivière and Galiana 2007; Whalen 2005). For example, tobacco (*Nicotiana tabacum*) develops enhanced resistance to *Phytopthora parasitica* during the transition to flowering. Enhanced resistance in reproductive-stage tobacco plants is associated with PATHOGENESIS-RELATED 1 (PR1) accumulation and cytotoxic activity in the apoplast (Hugot et al. 1999). In rice (*Oryza sativa*), the onset of developmentally regulated resistance to *Xanthomonas oryzae* pv. *oryzae* occurs during the vegetative phase (Mazzola et al. 1994). In part this involves the interaction of the rice *Xa*-*21* resistance gene product with the *X. oryzae* Ax21 effector (Lee et al. 2009; Mazzola et al. 1994).

*Arabidopsis* salicylic acid (SA) accumulation mutants such as *sid2* (*salicylic acid induction deficient 2*), *eds1* (*enhanced disease susceptibility 1*), *eds5/sid1*, and *pad4* (*phytoalexin deficient 4*) are defective for the ARR response (Cameron and Zaton 2004; Carviel et al. 2009; Kus et al. 2002) indicating that SA accumulation is important during ARR. In addition, the ARR-defective *iap1*-*1* (*important for the ARR pathway 1*-*1*) mutant accumulates little SA in response to *Pst* (Carviel et al. 2009). The role of SA in defense signaling is well-documented (Reviewed in Vlot et al. 2009), however, the SA-signaling mutant *npr1*-*1* (*non*-*expressor of PR1*) shows a wild-type ARR response suggesting that SA may not play a conventional defense-signaling role during ARR (Kus et al. 2002). Moreover, in plants undergoing an ARR response SA accumulates in the intercellular space of leaves (Cameron and Zaton 2004). Based on these data we propose that SA acts as an antimicrobial agent during ARR. Consistent with this hypothesis, it was shown that intercellular washing fluids of mature plants undergoing ARR, as well as purified SA, have an antimicrobial effect on *Pst* in vitro (Cameron and Zaton 2004).

As a facultative long-day plant, *Arabidopsis* flowers later in short days than in long days (Gregory and Hussey 1953). We previously observed that in both short- and long-day-grown Col-0, ARR onset is associated with the floral transition at approximately 6 weeks post-germination (wpg) in short days and four wpg in long days (Rusterucci et al. 2005). Several studies indicate that regulatory elements are shared between disease resistance and flowering pathways in *Arabidopsis*, including SA, which in addition to its role in disease resistance, also plays a role in flowering-time control (Reviewed in Rivas-San Vicente and Plasencia 2011). For example, evidence suggests that the SUMO and ubiquitin E3 ligases SIZ1 and PUB13 modify proteins that affect SA-related defense responses and flowering-time (Jin et al. 2008; Lee et al. 2006; Liu et al. 2012). Moreover, Wang et al. (2011) found that the putative acetylornithine transaminase encoded by *WIN3* acts together with other SA regulatory proteins such as NPR1 to control cell death, disease resistance, and flowering time. SA accumulation mutants such as *sid2* have been observed to flower later than wild type and also produce greater rosette leaf biomass and seed yield (Abreu and Munné-Bosch 2009; Martínez et al. 2004). However, this has not been observed for the ARR-defective and SA accumulation-deficient mutant *iap1*-*1* (Carviel et al. 2009).

**Fig. 1:**
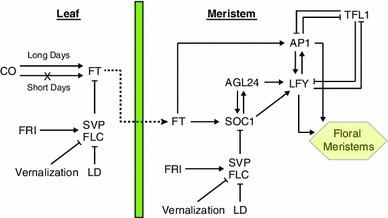
Simplified representation of flowering-time regulation adapted from Amasino (2010) and Fornara et al. (2010). The FLC-SVP complex represses *FT* in the leaf and *SOC1* in the meristem and is regulated by FRI, vernalization, and the components of the autonomous pathway (e.g., LD). In short days *FT* expression remains low, whereas in long days *FT* is upregulated by CO and FT protein moves via the phloem (*dashed line*) from the leaf to the shoot apical meristem where it directly upregulates *SOC1* and *AP1*. SOC1 upregulates *AGL24* and *LFY*. LFY and AP1 are responsible for the production of floral meristems and are repressed by TFL1

The timing of the floral transition is highly regulated and is controlled by several major pathways that respond to environmental and endogenous stimuli. These pathways converge on a group of integrator genes that regulate the floral meristem-identity genes responsible for floral organ development at the shoot apical meristem (SAM). Figure 1 is a schematic diagram of flowering-time regulation in *Arabidopsis* that is limited to the flowering-time genes that are relevant to this study. Environmental cues that affect flowering include day length (photoperiod pathway), prolonged periods of cold (vernalization pathway), and ambient temperature. Other pathways respond to endogenous stimuli, for example, the autonomous, gibberellin, and ageing pathways (Reviewed in Amasino 2010; Simpson and Dean 2002).

FLOWERING LOCUS T (FT) is a floral integrator that links day-length perception in the leaves to floral induction at the SAM. During long days, the CONSTANS (CO) transcription factor accumulates in the phloem companion cells of leaves and upregulates *FT* transcription (An et al. 2004; Ayre and Turgeon 2004; Suárez-López et al. 2001; Yanovsky and Kay 2002). FT moves from the leaves via the phloem to the SAM (Corbesier et al. 2007; Jaeger and Wigge 2007) where it upregulates another floral integrator *SUPRESSOR OF OVEREXPRESSION OF CO 1* (*SOC1*) (Michaels et al. 2005; Yoo et al. 2005) and the floral meristem-identity gene *APETALA 1* (*AP1*) (Abe et al. 2005; Wigge et al. 2005). *SOC1* participates in a positive feedback loop with *AGAMOUS*-*LIKE 24* (*AGL24*) to up-regulate the *LEAFY* (*LFY*) floral meristem identity gene (Lee et al. 2008; Liu et al. 2008).

*AP1* and *LFY* are involved in the production of floral meristems (Weigel et al. 1992, Irish and Sussex 1990) and are repressed by TERMINAL FLOWER 1 (TFL1), which is responsible for maintenance of the indeterminate inflorescence meristem (Alvarez et al. 1992; Liljegren et al. 1999; Schultz and Haughn 1993; Shannon and Meeks-Wagner 1991; Shannon and Meeks-Wagner 1993). Evidently, TFL1 also represses flowering in vegetative plants since *tlf1* mutants flower early compared to wild type (Schultz and Haughn 1993; Shannon and Meeks-Wagner 1991). FT and SOC1 also incorporate signals from the vernalization and autonomous flowering pathways. *Arabidopsis* accessions possessing a dominant *FRIGIDA* (*FRI*) allele usually require a vernalization treatment before they become competent to flower (Lee and Amasino 1995; Lee et al. 1993). FRI confers a vernalization requirement by upregulating the floral repressor and MADS-box transcription factor *FLOWERING LOCUS C* (*FLC*) (Michaels and Amasino 1999). FLC forms a high-molecular-weight complex with another MADS-box transcription factor SHORT VEGETATIVE PHASE (SVP), and represses flowering by directly binding to regulatory regions of *FT* and *SOC1* in both leaf and SAM tissue (Helliwell et al. 2006; Lee et al. 2007; Li et al. 2008; Searle et al. 2006). Vernalization confers reproductive competence by derepressing *SOC1* and *FT* through epigenetic silencing of *FLC* (Bastow et al. 2004). Autonomous pathway mutants such as *ld*-*1* (*luminidependens*-*1*) are late flowering and exhibit increased expression of *FLC* and *SVP*, suggesting that the autonomous pathway is responsible for controlling the levels of FLC and SVP (Li et al. 2008; Michaels and Amasino 1999; Michaels and Amasino 2001; Sheldon et al. 1999).

The aim of this study is to determine whether the floral transition plays a role in regulating ARR competence. To do this we asked whether the association between flowering and ARR is maintained in flowering-time mutants. To examine the role of photoperiod-induced flowering in ARR onset we separated photoperiod-induced flowering from long-day growth conditions. Our results suggest that in both short- and long-day conditions, flowering is not the developmental cue that initiates ARR competence. We present evidence that *SVP* is required for ARR and propose that in short-day conditions the development of a minimum number of rosette leaves is necessary to initiate ARR competence.

## Materials and methods

### Plant material and growth conditions

Wild-type Columbia (Col-0) and Wassilewskija (Ws-2) accessions were used. All mutants used in this study were in the Columbia background. Mutants that were previously confirmed to be ARR-defective were *sid2*-*1* (C. Nawrath, University of Fribourg, Fribourg, Switzerland) and *iap1*-*1* (described in Carviel et al. 2009). Flowering-time mutants *ld*-*1* (CS3127), *svp*-*31* (SALK_026551C), and *tfl1*-*14* (CS6238) were obtained from the Arabidopsis Biological Resource Centre, Ohio State University, Columbus OH, USA (Alonso et al. 2003). *co*-*9*, *ft*-*10*, *FRI* ^+^, and *FRI* ^+^ *flc*-*3* were supplied by R. Amasino (University of Wisconsin-Madison, WI, USA). *soc1*-*2* was supplied by I. Lee (Seoul National University, Seoul, Korea). *35S:miR156* was obtained from S. Poethig (University of Pennsylvania, PA, USA). *svp*-*32* (SALK_072930) was obtained from J. H. Ahn (Korea University, Seoul, Korea; Lee et al. 2007). Seeds were surface-sterilized and stratified at 4 °C for 2 days before sowing on MS media where they germinated under constant light at 22 °C. Seedlings were transplanted to soil (Sunshine Mix #1) hydrated with 1 g L^−1^ 20–20–20 all-purpose fertilizer approximately 1 week later. Plants were grown in short days unless otherwise specified. Short days consisted of 9 h light, and long days consisted of 16 h light. Light intensity was maintained at approximately 150 μE m^−2 ^s^−1^ and temperature at 23 °C. Short-day growth chambers had added humidity (75–85 % relative humidity) whereas the long-day chamber did not (50–70 % relative humidity). Rosette leaves that were large enough to be resolved without magnification were scored to determine rosette leaf number.

### Bacterial growth, inoculation, and quantification

Virulent *P. syringae* pv. *tomato* (*Pst*) strain DC3000 (pVSP61) was used in all experiments (A. Bent, University of Wisconsin-Madison, WI, USA). Bacteria were cultured in King’s B media with shaking at room temperature to exponential phase (OD_600_ = 0.2–0.6) and then diluted to 10^6^ colony forming units ml^−1^ in 10 mM MgCl_2_. Inoculum was pressure-infiltrated into the abaxial side of leaves using a needle-less syringe. Isolation and quantification of *Pst* at 3 days post-inoculation was performed as described previously (Kus et al. 2002).

### Analysis of gene expression by RT-PCR

Leaf tissue was harvested in the evening (end of photoperiod), flash-frozen in liquid nitrogen, and stored at −80 °C until further use. RNA was isolated using Sigma TRI Reagent according to the manufacturer’s instructions. Residual DNA was degraded using TURBO DNase (Life Technologies) prior to RNA quantification. First-strand cDNA synthesis was carried out using SuperScript III reverse transcriptase (Life Technologies). PCR primers used to amplify *FT* transcripts were: 5′-TAAGCAGAGTTGTTGGAGACG and 5′-TCTAAAGTCTTCTTCCTCCGCAG (Jang et al. 2009). Primers used to amplify *ACTIN1* transcripts were: 5′-GGCGATGAAGCTCAATCCAAACG and 5′-GGTCACGACCAGCAAGATCAAGACG. Twenty-eight PCR cycles were used for both *FT* and *ACTIN1*.

### Statistical analysis

Statistically significant differences in bacterial densities and average rosette leaf numbers were determined by ANOVA. To account for unequal variance in the means the bacterial density data were transformed prior to analysis (log or square root transformation). Tukey’s HSD post hoc test was used for pair-wise comparisons (*p* < 0.01). All tests were performed using IBM SPSS Statistics 20.

## Results

### ARR onset does not coincide with the floral transition in early- and late-flowering plant lines

**Fig. 2:**
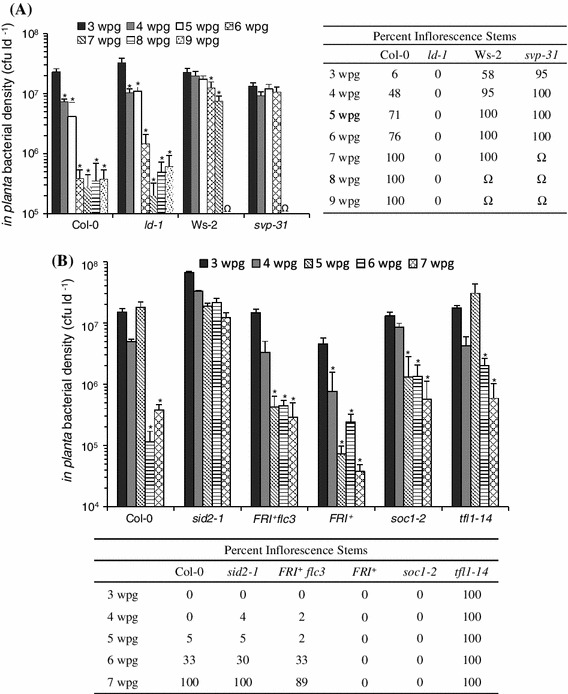
ARR responses of various flowering-time mutants. **a** Col-0, *ld*-*1*, Ws-2 and *svp*-*31* were grown in short days and tested for ARR each week between three and nine wpg. Plants were inoculated with 10^6^ cfu ml^−1^ virulent *Pst* (DC3000) and bacterial levels were quantified 3 days later. Data are presented as the mean of three biological replicates. *Error bars* indicate standard deviation. *Asterisk* denotes significant differences relative to three wpg plants of the same genotype according to Tukey’s HSD (*p* < 0.01). Each week at least 12 plants of each genotype were assessed for visible inflorescence stems. *Values* represent the percentage of plants with visible inflorescence stems. *Ω* indicates the onset of senescence at which point further testing was not possible. Each genotype was tested at least three times with similar results **b** Col-0, *sid2*-*1*, *FRI* ^+^ *flc*-*3*, *FRI* ^+^, *soc1*-*2*, and *tfl1*-*14* were grown in short days and tested for ARR each week between three and seven wpg. Plants were inoculated with 10^6^ cfu ml^−1^ virulent *Pst* (DC3000) and bacterial levels were quantified 3 days later. Data are presented as the mean of three biological replicates. *Error bars* indicate standard deviation. *Asterisk* denotes significant differences relative to three wpg plants of the same genotype according to Tukey’s HSD (*p* < 0.01). Each week at least 12 plants of each genotype were assessed for visible inflorescence stems. *Values* represent the percentage of plants with visible inflorescence stems. This experiment was performed twice with similar results

If the transition to flowering acts as a developmental cue to initiate ARR competence, we should observe delayed ARR onset in late-flowering mutants and early ARR onset in early-flowering mutants. To test this hypothesis, wild-type Col-0, early-flowering *svp*-*31* (Hartmann et al. 2000), late-flowering *ld*-*1* (Rèdei 1962), and the early-flowering Ws-2 accession (Giakountis et al. 2010) were analyzed. *In planta* bacterial levels were monitored from 3 to 9 weeks post germination (wpg) by inoculating with virulent *Pst* (10^6^ cfu ml^−1^) followed by isolation and quantification of *in planta* bacteria 3 days later. The transition to flowering was approximated by counting the percentage of plants with visible inflorescence stems each week. In this experiment Col-0 flowered earlier than typically observed when grown under short-day conditions, such that 48 % of plants had visible inflorescence stems at four wpg, 71 % at five wpg, and 76 % at six wpg (Fig. 2a), instead of 0 % at 4 wpg, 5 % at 5 wpg and 33 % at 6 wpg (Fig. 2b). A power outage that interrupted the photoperiod regimen in week 3 exposed the plants to a displaced short day. Displaced short days have been shown to cause early flowering in *Arabidopsis* (Corbesier et al. 1996). While *ld*-*1* did not produce inflorescence stems during the experiment, 95 % of *svp*-*31* produced inflorescence stems by three wpg. Ws-2 also made the transition to flowering earlier than Col-0, with 58 % of plants showing inflorescence stems at three wpg, and 95 % at four wpg.

Col-0 became increasingly resistant to *Pst* between three and six wpg (Fig. 2a). Young (three wpg) plants supported high levels of *Pst* (2.3 × 10^7^ cfu ld^−1^), while 4- and 5-week-old plants supported modestly reduced levels (7.4 × 10^6^ and 4.1 × 10^6^ cfu ld^−1^) and mature plants (six to nine wpg) supported low levels of *Pst* (<4.0 × 10^5^ cfu ld^−1^). There was a 60-fold decrease in *Pst* between 3- and 6-week-old plants, indicating that Col-0 was fully ARR-competent at six wpg. Late-flowering *ld*-*1* supported *Pst* levels similar to Col-0 between three and five wpg. At six wpg *ld*-*1* *Pst* levels dropped to 1.5 × 10^6^ cfu ld^−1^ (22-fold less than at three wpg) indicative of moderate ARR. At seven, eight, and nine wpg *ld*-*1* displayed a robust ARR response (97-fold reduction in *Pst* levels between three and seven wpg). Early-flowering *svp*-*31* supported high *Pst* densities (>1.0 × 10^7^ cfu ld^−1^) from three to six wpg, remaining ARR-incompetent. At seven wpg, older *svp*-*31* leaves began to senesce as indicated by yellowing and necrosis, therefore these plants were not tested beyond six wpg. A second mutant allele, *svp*-*32*, also flowered early and was found to be ARR-defective (Fig. S2). The early-flowering Ws-2 accession also supported high *Pst* densities (>7.0 × 10^6^ cfu ld ^−1^) at all ages.

Although somewhat delayed compared to Col-0, the *ld*-*1* mutant displayed a robust ARR response even in the absence of flowering, suggesting that the floral transition is not required for ARR competence. In addition, early-flowering does not elicit an early ARR response since *svp*-*31*, *svp*-*32* and Ws-2 flowered early but did not display ARR at the time of the floral transition or at any time thereafter. Therefore, the floral transition does not appear to act as a developmental cue for ARR competence.

### Photoperiod-induced flowering does not elicit ARR competence

**Fig. 3:**
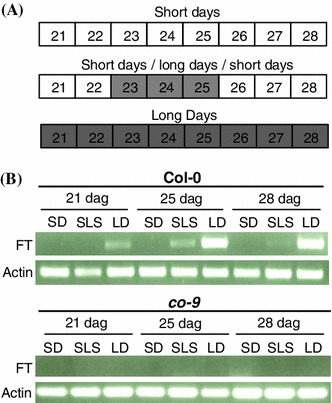
Short-day-grown plants express *FT* after exposure to three long days. **a** Schematic representation of the three photoperiod regimens. *White bars* indicate short days (9 h light), *dark bars* indicates long days (16 h light). *Numbers* indicate days after-germination. **b** *FT* and *ACTIN* expression measured by RT-PCR in leaf tissue of Col-0 and *co*-*9* plants grown in short days (SD), short days/long days/short days (SLS), or long days (LD). Tissue was collected in the evening on 21, 25, and 28 days after-germination (dag)

To support the hypothesis that the floral transition does not confer ARR competence we used short day/long day shift experiments to elicit precocious flowering in young, short-day-grown Col-0 followed by an assay for ARR competence. Brief exposure of short-day-grown plants to inductive (long-day) photoperiods activates the photoperiod pathway and initiates the transition to flowering (Corbesier et al. 2007; Imaizumi et al. 2003). Eliciting early flowering in wild-type plants has the advantage of avoiding possible pleiotropic effects of mutations in flowering-time genes. Col-0 was grown under three different photoperiod regimens and tested for ARR competence at four wpg. Photoperiod regimens consisted of either short days, long days, or short days plus three long days followed by return to short days (Fig. 3a). All long-day-grown Col-0 had visible inflorescence stems at four wpg, whereas short-day-grown and photoperiod-induced short-day-grown Col-0 did not. To determine whether photoperiod-induced short-day-grown plants had made the transition to flowering, RT-PCR was used to measure *FT* expression in leaf tissue taken at time points spanning the induction period (Fig. 3b). *FT* expression was detected in the leaves of photoperiod-induced short-day-grown Col-0 at the end of the third long day, indicating that the photoperiod pathway had been activated. *FT* expression was consistently detected in the leaves of long-day-grown Col-0 and was not detected in short-day-grown plants.

**Fig. 4:**
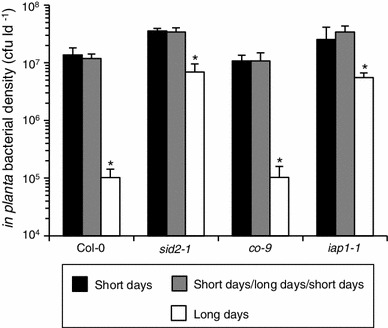
Photoperiod-induced flowering does not elicit ARR. Col-0, *sid2*-*1*, *co*-*9*, and *iap1*-*1* were grown in short days, short days/long days/short days, or long days and were inoculated with 10^6^ cfu ml^−1^ virulent *Pst* (DC3000) at four wpg. Bacterial levels were quantified 3 days later and are presented as the mean of three biological replicates. *Error bars* indicate standard deviation. *Asterisk* indicates significant differences as determined by ANOVA (Tukey’s HSD, *p* < 0.01). This experiment was performed twice with similar results

Consistent with previous experiments (Rusterucci et al. 2005), at four wpg long-day-grown Col-0 supported few disease symptoms and low bacterial levels whereas short-day-grown Col-0 was susceptible, supporting 125-fold higher *Pst* levels than long-day-grown plants (Fig. 4). This indicates that long-day-grown Col-0 was ARR-competent at four wpg whereas short-day-grown plants were not. Photoperiod-induced short-day-grown Col-0 supported high *Pst* densities at four wpg (1.2 × 10^7 ^cfu ld^−1^), similar to short-day-grown Col-0 (1.4 × 10^7 ^cfu ld^−1^), therefore these plants were not competent for ARR at four wpg. This confirms our previous conclusion that the floral transition does not confer ARR competence.

### CONSTANS is not required for ARR in long days

Since ARR onset occurs earlier in long-day conditions we wanted to determine whether the transition to flowering, or a different developmental event accelerated in long days, elicits ARR competence. For example, the vegetative phase change from juvenile to adult vegetative stages occurs earlier in long-day-grown plants (Chien and Sussex 1996; Willmann and Poethig 2005) and could be associated with ARR competence. To separate flowering from other developmental changes that might act as a switch for ARR-competence in long days we tested *co* mutants which flower late in long days (Koornneef et al. 1991; Putterill et al. 1995) because *FT* is no longer up-regulated by CO in a photoperiod-dependent manner (Kardailsky et al. 1999; Kobayashi et al. 1999; Samach et al. 2000). If the transition to flowering is the cue for ARR competence in long days, then long-day-grown *co* mutants should have delayed ARR compared to Col-0 (ARR at four wpg). To test this hypothesis the *co*-*9* mutant was grown in three different photoperiod regimens and tested for ARR competence at four wpg as described previously. In all three photoperiod regimens *co*-*9* lacked inflorescence stems and detectable *FT* expression throughout the experiment (Fig. 3b), indicating that the photoperiod pathway was not activated. Short-day-grown and photoperiod-induced short-day-grown *co*-*9* supported high *Pst* levels (1.1 × 10^7^ cfu ld^−1^), whereas long-day-grown *co*-*9* supported low levels of *Pst* (1.0 × 10^5^ cfu ld^−1^; Fig. 4). A 110-fold decrease in *Pst* levels in long-day-grown compared to short-day-grown *co*-*9* is indicative of a robust ARR response in long-day-grown plants. In long-day-grown *co*-*9* mutants ARR occurs in the absence of flowering, demonstrating that photoperiod-induced flowering is not required for the establishment of ARR competence in long-day conditions.

### ARR competence is associated with leaf number in short-day conditions

Our original hypothesis that the floral transition is the developmental cue for ARR competence was not supported, therefore other developmental events that might act as a switch to initiate ARR-competence were considered. The early-flowering plant lines (*svp*-*31*, *svp*-*32*, and Ws-2) were ARR-defective and produced few rosette leaves (Table 1; Figs. S1 and S2). The SAM switches from production of vegetative to reproductive structures during the transition to flowering, therefore the timing of the floral transition affects vegetative growth (rosette leaf number) such that early-flowering plant lines produce fewer rosette leaves than wild type (Hempel and Feldman 1994; Koornneef et al. 1991). It has been suggested that the timing of some developmental events may be influenced by rosette leaf number (McDaniel et al. 1992; Poethig 1990; Schultz and Haughn 1993) and given that the early-flowering plant lines examined thus far produced few rosette leaves and were ARR-defective, we hypothesized that the development of a minimum number of rosette leaves might initiate ARR competence. To assess whether ARR competence is associated with leaf number we analyzed the early-flowering *tfl1*-*14* mutant (Schultz and Haughn 1993) because it produced more rosette leaves than *svp*-*31*, *svp*-*32*, and Ws-2 (Table 1; Figs. S1 and S2). In these experiments plants were assessed for ARR competence and average rosette leaf number between three and seven wpg.

**Table 1:** ARR onset and leaf number of short-day-grown plants

	Flowering-time phenotype^a^	ARR onset	Average rosette leaves^c^ at ARR onset
*ARR-competent plant lines*			
Col-0	Wild-type	6 wpg	33.9 ± 2.8
*FRI* ^+^ *flc*-*3*	Wild-type	5 wpg	26.4 ± 2.8^b^
*FRI* ^+^	Late	5 wpg	27.2 ± 1.3^b^
*ld*-*1*	Late	6 wpg	32.7 ± 2.6
*soc1*-*2*	Late	5 wpg	27.0 ± 2.1^b^
*tfl1*-*14*	Early	6 wpg	28.3 ± 1.7
*ARR*-*defective plant lines*			
Ws-2	Early	na	22.8 ± 4.3^c,d^
*svp*-*31*	Early	na	21.7 ± 3.8^c,d^

ARR onset and leaf number for experiment presented in Fig. 2a, b. Average rosette leaves for Col-0 from experiment in Fig. 2b

^a^Relative to wild-type Col-0

^b^Rosette leaf number is presented as the average ± standard deviation (n = 9)

^c^Significantly different from Col-0 (ANOVA, Tukey’s HSD, *p* < 0.01)

^d^For ARR-defective plant lines the maximum average rosette leaf number is presented

Col-0 made the transition to flowering between six and seven wpg (33 and 100 % inflorescence stems, respectively) and supported high *Pst* densities (≥5.0 × 10^6^ cfu ld^−1^) between three and five wpg (Fig. 2b). At six wpg *Pst* levels dropped to 1.2 × 10^5^ cfu ld^−1^, a 131-fold reduction compared to 3-week-old plants, indicative of a robust ARR response. Col-0 had a rosette leaf number of 33.9 ± 2.8 at six wpg (Table 1) for the experiment presented in Fig. 2b. Moreover, in five independent experiments, Col-0 produced an average of 34.7 ± 3.4 rosette leaves at ARR onset (6 wpg), making it possible to compare leaf number across experiments (Tables 1, 2). ARR-defective *sid2*-*1* supported high bacterial densities at all ages (>1.0 × 10^7^ cfu ld^−1^) and flowered at approximately the same time as Col-0 (30 and 100 % inflorescence stems at six and seven wpg respectively). At all ages *sid2*-*1* had a rosette leaf number similar to Col-0 (Fig. S1). This suggests that *sid2*-*1* is developmentally similar to Col-0 in terms of leaf number and is consistent with previous work suggesting that the *sid2*-*1* ARR defect is due solely to its inability to accumulate SA (Cameron and Zaton 2004). The transition to flowering occurred prior to three wpg in *tfl1*-*14*, as 100 % of plants had inflorescence stems by this time (Fig. 2b). *tfl1*-*14* supported high *Pst* levels at three and five wpg (>1 × 10^7 ^cfu ld^−1^) and a statistically insignificant decline at four wpg (4.2 × 10^6 ^cfu ld^−1^). At six wpg *Pst* levels in *tfl1*-*14* were reduced to 2.0 × 10^6 ^cfu ld^−1^, characteristic of a modest ARR response (9-fold reduction in *Pst* levels relative to three wpg). A more robust ARR response was observed at seven wpg (30-fold reduction in *Pst* levels compared to three wpg). This indicates that ARR occurs in the early-flowering *tfl1*-*14* mutant. At six wpg *tfl1*-*14* had a rosette leaf number of 28.3 ± 1.7, not significantly different from Col-0 (Table 1). The ARR-defective early-flowering plant lines *svp*-*31* and Ws-2 had rosette leaf numbers of 21.7 ± 3.8 and 20.9 ± 6.3 respectively at six wpg; significantly less than *tfl1*-*14* or Col-0. These results suggest that development of a minimum number of rosette leaves is necessary to initiate ARR competence in short-day-grown plants.

**Table 2:** ARR in 4-week-old Col-0 and *co*-*9* grown in different photoperiod regimens

	Photoperiod^a^	Floral transition^c^	ARR response^c^	Average rosette leaves^b,c^
Col-0	SD	–	−	23.1 ± 1.7
	SLS	+	−	24.3 ± 2.3
	LD	+	+	17.4 ± 2.0
*co*-*9*	SD	−	−	24.6 ± 2.0
	SLS	−	−	24.7 ± 1.5
	LD	−	+	29.1 ± 2.8

ARR response and leaf number for experiment presented in Fig. 4

^a^Short days (SD), long days (LD), or short days/long days/short days (SLS)

^b^Rosette leaf number is presented as the average ± standard deviation (n = 18)

^c^Measurements were taken at 4 weeks post-germination

The *ld*-*1* mutant displayed ARR in the absence of flowering, however, the ARR response was somewhat delayed compared to that of Col-0 (Fig. 2a). To test whether ARR is delayed in the absence of flowering, and to determine whether this could be explained in terms of leaf number, we analyzed two additional late-flowering lines; the *soc1*-*2* mutant (Borner et al. 2000) and a *FRI* ^+^ Col-0 line hereafter referred to as *FRI* ^+^ (Lee and Amasino 1995). *SOC1* integrates signals from multiple flowering pathways, therefore *soc1*-*2* mutants flower later than wild type (Borner et al. 2000). Wild-type Col-0 has recessive alleles of the *FRI* gene and as a result, flowers without vernalization (Johanson et al. 2000; Lee and Amasino 1995). A dominant *FRI* allele introgressed into the Col-0 background severely delays flowering in the absence of vernalization due to upregulation of the floral repressor *FLC* (Lee and Amasino 1995). *FRI* ^+^ and *soc1*-*2* were chosen primarily for their late-flowering phenotypes. Also, to our knowledge there is no evidence that they exhibit developmental phenotypes aside from late flowering (see discussion on *ld*-*1*), however, we also tested a *FRI* ^+^ *flc*-*3* line which flowers at the same time as wild-type Col-0 (Michaels and Amasino 1999) and therefore serves as a control for potential pleiotropic effects of the dominant *FRI* allele. For example, if *FRI* ^+^ had an ARR defect that was caused by its late-flowering phenotype, this defect should not be observed in *FRI* ^+^ *flc*-*3* which flowers at the same time as wild type. However, if *FRI* ^+^ had an ARR defect for a reason other than late flowering, then *FRI* ^+^ *flc*-*3* should display that same defect. As expected, neither *soc1*-*2* nor *FRI* ^+^ flowered during our experiments while *FRI* ^+^ *flc*-*3* flowered at approximately the same time as Col-0 (Fig. 2b). *soc1*-*2* supported high *Pst* levels at three and four wpg (>8.0 × 10^6 ^cfu ld^−1^), intermediate levels at five and six wpg (1.4 × 10^6 ^cfu ld^−1^) and lower levels at seven wpg (5.7 × 10^5 ^cfu ld^−1^; Fig. 2b). At five and six wpg there was a 10-fold reduction in *Pst* levels compared to three wpg, indicative of a moderate ARR response in *soc1*-*2*. By seven wpg this difference had increased to 23-fold lower levels of *Pst* compared to three wpg. s*oc1*-*2* had a rosette leaf number of 27.0 ± 2.1 at the time of ARR onset (Table 1). *FRI* ^+^ supported relatively high *Pst* levels at three wpg (4.5 × 10^6 ^cfu ld^−1^), intermediate levels at four wpg (7.7 × 10^5 ^cfu ld^−1^) and low levels between five and seven wpg (<1.2 × 10^5 ^cfu ld^−1^; Fig. 2b). There was a 63-fold decrease in *Pst* levels between three and five wpg, indicative of a robust and early ARR response. *FRI* ^+^ had a rosette leaf number of 27.2 ± 1.3 at the time of ARR onset (Table 1). Bacterial levels in *FRI* ^+^ *flc*-*3* were similar to those of *FRI* ^+^ and ARR was also first observed at five wpg (35-fold reduction in *Pst* relative to three wpg; Fig. 2b). *FRI* ^+^ *flc*-*3* produced a similar rosette leaf number to *FRI* + at the time of ARR onset (Table 1). Neither *FRI* ^+^ nor *soc1*-*2* flowered during the experiment, but both displayed ARR, further supporting the conclusion that flowering is not necessary for ARR competence. The rosette leaf number of *FRI* + and *soc1*-*2* at the time of ARR onset was significantly lower than Col-0 but still higher than the maximum reached by the ARR-defective plant lines *svp*-*31*, *svp*-*32*, and Ws-2 (Table 1, Fig. S2). The observation that one late-flowering mutant (*ld*-*1*) had delayed ARR while two other late-flowering plant lines (*FRI* ^+^, *soc1*-*2*) and a wild-type flowering-time plant line (*FRI* ^+^ *flc*-*3*) had early ARR indicates that the timing of ARR onset varies between plant lines independently of the timing of the floral transition. Altogether the leaf number data presented in Table 1 is consistent with the hypothesis that development of a minimum number of rosette leaves is required for ARR competence in short-day-grown plants.

To determine whether our hypothesis of a minimum rosette leaf number requirement also applies to long-day-grown plants we analyzed rosette leaf number data collected during the short day/long day shift experiments described above. 4-week-old short-day-grown and photoperiod-induced short-day-grown Col-0 and *co*-*9* had low rosette leaf numbers (between 23 and 25; Table 2) and were ARR-incompetent at this time. This is consistent with our observations that short-day-grown plants remain ARR-incompetent until the production of approximately 30 rosette leaves (Table 1). At four wpg, short-day-grown and photoperiod-induced short-day-grown plants were either vegetative or just beginning the transition to flowering. In contrast, long-day-grown Col-0 made the transition to flowering at approximately three wpg (100 % of plants had inflorescence stems) and therefore had developed fewer rosette leaves (17.4 ± 2.0) than short-day-grown plants at four wpg. The observation that long-day-grown Col-0 was ARR-competent with so few rosette leaves is not consistent with the leaf number-ARR competence relationship observed for short-day-grown plants. This could indicate that the leaf number threshold for ARR competence is lower for plants grown in long days or alternatively, that ARR competence is regulated by a different mechanism in long-day-grown plants.

### *IAP1* and *SID2* are required for ARR in long days

*IAP1* and *SID2* are important components of the ARR response that occurs in short-day-grown plants (Carviel et al. 2009; Kus et al. 2002). Plants grown in long days display a similar but earlier ARR response (Rusterucci et al. 2005). To obtain clues as to whether the ARR pathway in short-day-grown plants shares components with the ARR pathway in long-day-grown plants, two mutants that are known to be ARR-defective in short-day conditions, *iap1*-*1* and *sid2*-*1*, were examined in three different photoperiod regimens as described previously. Short-day-grown and photoperiod-induced short-day-grown *iap1*-*1* and *sid2*-*1* all supported high levels of *Pst* (>1.0 × 10^7^ cfu ml^−1^) similar to Col-0 (Fig. 4). Long-day-grown *iap1*-*1* and *sid2*-*1* both supported lower *Pst* densities compared to their short-day-grown counterparts (5-fold reduction), however, these plants were still susceptible as indicated by high *Pst* levels (50- to 70-fold higher than long-day-grown Col-0) and characteristic disease symptoms (data not shown), indicative of a defective ARR response. The lower *Pst* levels in long-day-grown plants were probably due to lower humidity in the long-day chamber (60 %) compared to the short-day chamber (80 %), since high humidity enhances *in planta* *Pst* growth (Agrios 2005). Both *iap1*-*1* and *sid2*-*1* had a similar rosette leaf number to Col-0 in all three photoperiod regimens (data not shown). It has been reported that *sid2*-*1* flowers later than Col-0 (greater total leaf number at bolting; Martínez et al. 2004), however this is not observed in our experiments perhaps due to differences in plant growth conditions (day length, light quantity and humidity differences). Taken together, these data suggest that these SA-deficient mutants are developmentally similar to Col-0 in terms of leaf number, and the capacity to accumulate SA is required for ARR in *Arabidopsis* grown in long-day as well as short-day photoperiods.

## Discussion

### ARR competence is not associated with flowering in short- or long-day conditions

Previously we demonstrated that ARR competence is associated with the floral transition in Col-0 (Rusterucci et al. 2005). Here we sought to determine if the transition to flowering is responsible for initiating ARR competence by separating the transition to flowering from other developmental events that occur as plants age. To do this, the ARR phenotypes of mutants with three classes of flowering-time phenotype (early, late, and wild-type) were examined under short-day conditions. Overall there was no clear relationship between flowering time and the timing of ARR onset, with all ARR-competent plant lines displaying ARR between five and six wpg irrespective of flowering time. For example, late-flowering plant lines (*ld*-*1*, *FRI* ^+^, *and soc1*-*2*) displayed ARR at approximately the same time as Col-0 even though they did not flower during our experiments. This suggests that the floral transition is not required to initiate an ARR-competent state. Of the four plant lines that flowered early, *svp*-*31*, *svp*-*32*, and Ws-2 were defective for ARR and *tfl1*-*14* displayed a moderate ARR response. Even though *tfl1*-*14* had completed the floral transition by three wpg, ARR was not observed until six wpg, suggesting that early flowering does not initiate early ARR. The observation that *svp*-*31*, *svp*-*32*, and Ws-2 were ARR-defective further demonstrates that the floral transition is not involved in the initiation of ARR competence and led us to hypothesize that development of a minimum rosette leaf number is required to initiate ARR competence since *svp*-*31*, *svp*-*32*, and Ws-2 produced significantly fewer rosette leaves than either *tfl1*-*14* or Col-0. The fact that *tfl1*-*14* produced more rosette leaves than *svp*-*31*, *svp*-*32*, and Ws-2 is counter-intuitive since *tfl1*-*14* appeared to flower slightly earlier than *svp*-*31*, *svp*-*32*, and Ws-2 and would therefore be expected to have a lower maximum rosette leaf number. This difference could be explained by a higher leaf initiation rate in *tfl1*-*14* or a lower leaf initiation rate in *svp*-*31*, *svp*-*32*, and Ws-2 although it has previously been shown that *tfl1* mutants initiate leaves at a rate similar to Col-0 (Shannon and Meeks-Wagner 1991). Another explanation is that *tfl1*-*14* continued to produce rosette leaves after the transition to flowering, although this is inconsistent with the currently accepted model of organ development in reproductive-stage *Arabidopsis* which indicates that rosette leaves are not produced after the floral transition (Hempel and Feldman 1994).

To support the conclusion that the floral transition does not initiate ARR competence we looked at the ARR response of short-day-grown Col-0 that were forced to flower early by photoperiod-induced transient expression of *FT* (exposure to three long days). This treatment initiated the floral transition by four wpg as demonstrated by expression of *FT*, but did not elicit ARR competence suggesting that photoperiod-induced flowering is not sufficient for the onset of ARR competence in 4-week-old plants. This is consistent with the ARR defects observed in short-day-grown early-flowering plant lines and confirms that an early floral transition does not initiate ARR competence.

In *Arabidopsis* the floral transition occurs earlier in long days than in short days (Gregory and Hussey 1953). Since ARR onset also occurs earlier in long days and at approximately the same time as the transition to flowering, we suspected that the transition to flowering was the cue for ARR competence (Rusterucci et al. 2005). While this does not appear to be true for short-day-grown plants, we tested whether this might be the case for long-day-grown plants. Long-day-grown *co*-*9* mutants are delayed in photoperiod-induced flowering (Koornneef et al. 1991; Putterill et al. 1995) and remained vegetative at four wpg but still displayed a robust ARR response, similar to long-day-grown Col-0. This suggests that photoperiod-induced flowering is not required for the onset of ARR competence in long-day-grown plants. While it appears that development of a minimum rosette leaf number may initiate ARR competence in short-day-grown plants, the same relationship was not observed for long-day-grown plants since long-day-grown Col-0 displayed ARR at a rosette leaf number similar to short-day-grown, ARR-defective *svp*-*31*, *svp*-*32*, and Ws-2. This could indicate that the minimum leaf number requirement for ARR competence is lower in long-day-grown plants or that ARR in long-day-grown plants is regulated by a different mechanism altogether.

### Vegetative phase change and ARR competence

Another consideration is that the vegetative phase change could be involved in the regulation of ARR competence. The central regulator of the vegetative phase change, miRNA156, targets members of the *SQUAMOSA PROMOTER BINDING PROTEIN*-*LIKE* (*SPL*) family, which have been shown to contribute to the onset of adult and reproductive phase characteristics (Schwarz et al. 2008; Usami et al. 2009; Wang et al. 2008; Wu et al. 2009; Wu and Poethig 2006). In wild-type plants *miR156* levels decrease over time, leading to a gradual de-repression of *SPL* genes and transition to the adult vegetative phase. Overexpression of *miR156* in 35S:miR156 plants causes the juvenile phase to be dramatically prolonged; with plants producing 90 ± 1.3 juvenile leaves whereas Col-0 produces 7.5 ± 0.7 (Wu et al. 2009). Preliminary results from our lab indicate that short-day-grown *35S:miR156* plants exhibit ARR at six wpg (data not shown), suggesting that the prolonged manifestation of juvenile characteristics does not delay the onset of ARR competence.

Many mutations in flowering-time genes also affect the timing of the vegetative phase change. Alternatively, some mutations alter the timing of either the vegetative phase change or transition to flowering without affecting the other (Telfer et al. 1997; Willmann and Poethig 2005). Interestingly, ARR-defective *svp*-*31*, *svp*-*32*, and Ws-2 undergo an earlier vegetative phase change relative to Col-0 (Hartmann et al. 2000; Telfer et al. 1997) whereas ARR-positive *tfl1*-*14* undergoes the vegetative phase change normally (Telfer et al. 1997). Although this might suggest that an early vegetative phase change is associated with ARR incompetence, long-day-grown Col-0 also undergoes an early vegetative phase change (Chien and Sussex 1996), and this does not result in an ARR defect. This suggests that the timing of the vegetative phase change does not regulate ARR competence, however, a more detailed analysis is required to fully address this question.

### Timing of ARR onset differs between some plant lines

While some plant lines showed early ARR responses, others exhibited delayed ARR, such that robust resistance was not observed until seven wpg (*ld*-*1*) or only moderate responses were observed at six or seven wpg (*tfl1*-*14*, *soc1*-*2*). These differences had no obvious relationship with flowering-time. Instead it may be that some of the mutations that affect flowering time have pleiotropic effects. For example, autonomous pathway genes such as *LD* are believed to be involved in processes such as chromatin modification and RNA metabolism, and as a result, likely function in aspects of plant development other than flowering-time (Amasino 2010). This proposition is supported by observations of lethality or severe growth and developmental defects in various autonomous pathway mutants (Henderson et al. 2005; Koornneef et al. 1998; Veley and Michaels 2008). Variation in the timing of ARR could also result from differences in the genetic background of various plant lines used in this study (i.e., polymorphisms that are independent of mutations in flowering-time genes). Although all mutants used were in the Columbia background, whole-genome resequencing studies have revealed that different strains of Columbia can harbour thousands of unique polymorphisms (Ossowski et al. 2008). While many of these observed differences could reflect errors in the reference genome, the same group later showed that the rate of spontaneous mutation accumulation is much higher than previously thought (Ossowski et al. 2010). This implies that in some cases mutant lines may possess many genetic differences from wild-type controls (Santuari and Hardtke 2010).

We have demonstrated that the floral transition can be separated from ARR competence in both short- and long-day-grown plants. Therefore, the floral transition is not the developmental cue for ARR competence. Instead, vegetative development of a minimum numbers of leaves appears to be important for ARR competence in short-day grown *Arabidopsis*.

## Electronic supplementary material

Below is the link to the electronic supplementary material.

Supplementary material 1 (PDF 91 kb)
