# Supplementary material for: The floral transition is not the developmental switch that confers competence for the Arabidopsis age-related resistance response to Pseudomonas syringae pv. tomato
Source: Plant Mol Biol. 2013 May 31;83(3):235–46. doi: 10.1007/s11103-013-0083-7 (PMC3777159; doi:10.1007/s11103-013-0083-7)
Supplement: Supplementary file 1 — Supplementary material 1 (PDF 91 kb) [file 11103_2013_83_MOESM1_ESM.pdf]

Plant Molecular Biology

The floral transition is not the developmental switch that confers competence for the *Arabidopsis* Age-Related Resistance response to *Pseudomonas syringae* pv. *tomato*

Daniel C. Wilson*, Philip Carella*, Marisa Isaacs*, Robin K. Cameron

Department of Biology, McMaster University
1280 Main St West, Hamilton, Ontario, Canada
L8S 4L8

Correspondance:
e-mail: rcamero@mcmaster.ca
phone: (905) 525-9140 ext. 26345

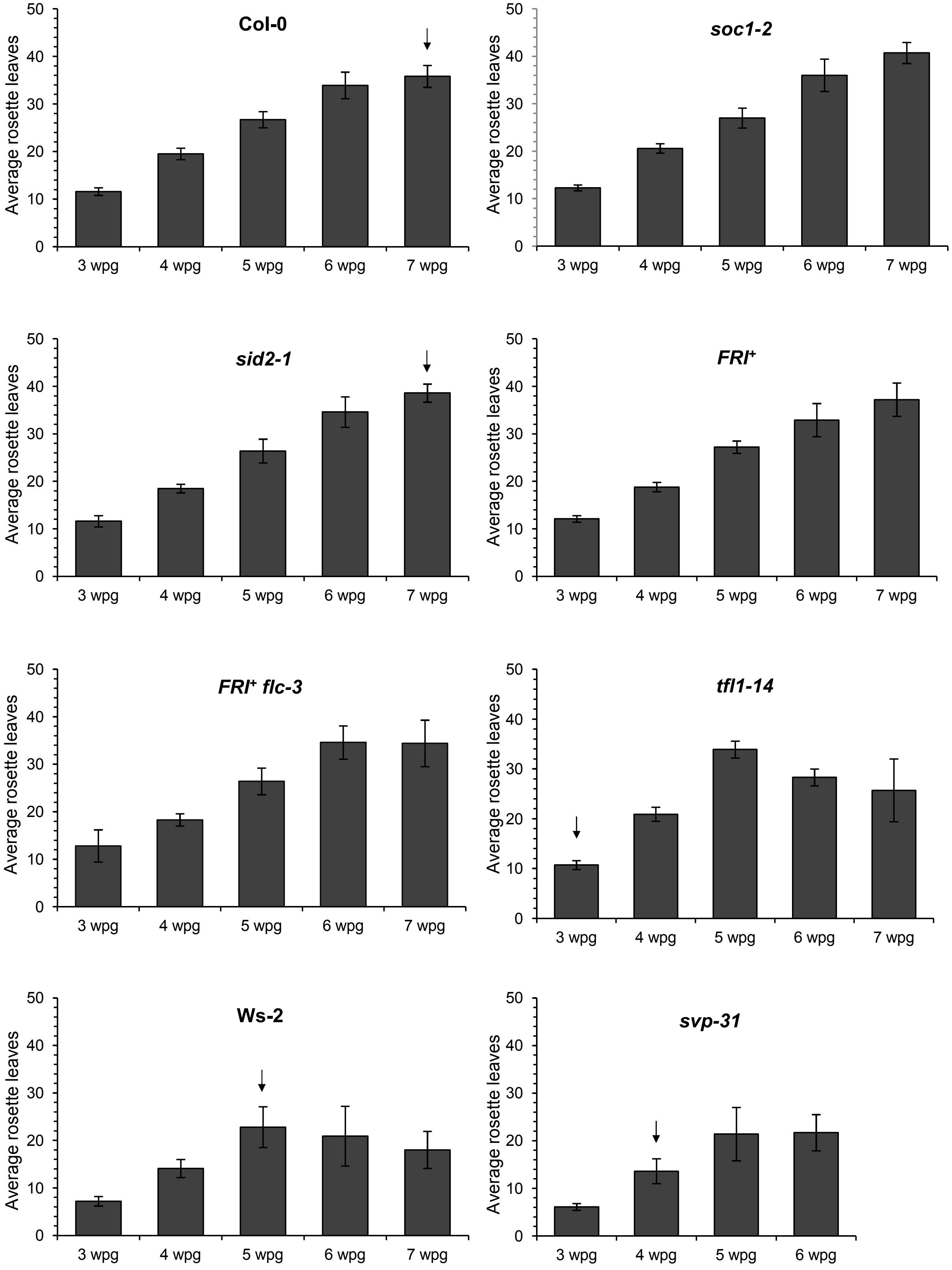

Col-0
soc1-2
sid2-1
FRI+
FRI+ flc-3
tfl1-14
Ws-2
svp-31
Average rosette leaves
0
10
20
30
40
50
3 wpg
4 wpg
5 wpg
6 wpg
7 wpg

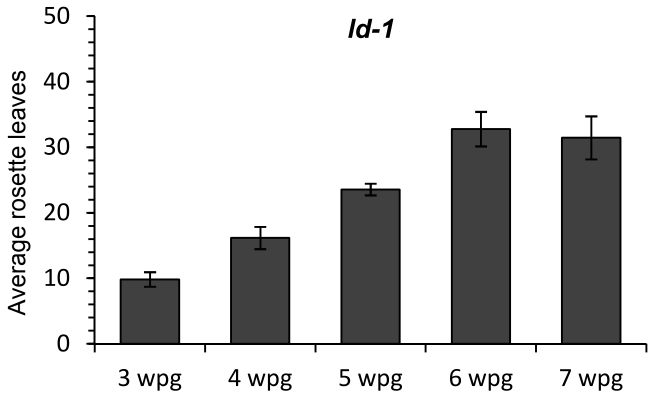

Supplementary Fig. 1 Average rosette leaf number between three and seven wpg. Each week a group of nine short-day-grown plants (Col-0, *sid2-1*, *FRI*$^{+}$ *flc-3*, *FRI*$^{+}$, Ws-2, *soc1-2*, *svp-31, tfl1-14, ld-1*) were analyzed for average rosette leaf number. For two of the three early-flowering lines (*svp-31*, *tfl1-14*) we observed that rosette leaf number continued to increase even after 100% of plants showed inflorescence stems. This is probably because the rosette leaf number values presented are an underestimate since only those leaves large enough to be resolved without magnification were counted at a given time. Therefore, several of the last rosette leaves were produced before the floral transition but were too small to count until one or two weeks after the floral transition. Arrows above bars mark the time at which 100% of plants showed inflorescence meristems.

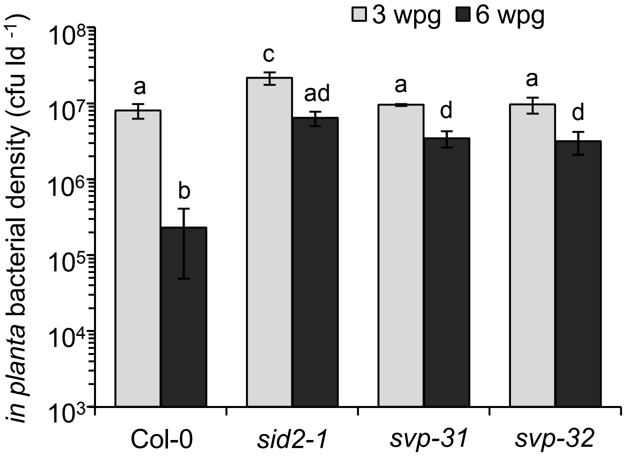

Percent inflorescence stems				
	Col-0	*sid2-1*	*svp-31*	*svp-32*
3 wpg	0	0	100	100
6 wpg	33	33	100	100

Average rosette leaf number				
	Col-0	*sid2-1*	*svp-31*	*svp-32*
3 wpg	13.8 ± 0.7	13.8 ± 0.8	9.8 ± 2.0	7.4 ± 0.7
6 wpg	36.4 ± 1.3	35.9 ± 1.2	14.1 ± 2.4	13.9 ± 2.6

Supplementary Fig. 2 *svp* mutants are defective for ARR. Col-0, *sid2-1, svp-31, and svp-32* were grown in short days and tested for ARR at three and six wpg. Plants were inoculated with 10^6 cfu ml^{-1} virulent *Pst* (DC3000) and bacterial levels were quantified three days later. Data are presented as the mean of three biological replicates. Error bars indicate standard deviation. Different letters indicate significant differences (Tukey's HSD [p<0.01]). Visible inflorescence stems and average rosette leaf number were scored at 3 and 6 wpg. Rosette leaf number values are presented as the average ± standard deviation (n=9). This experiment was performed twice with similar results.
